# The role of Melancholia in prostate cancer patients' depression

**DOI:** 10.1186/1471-244X-11-201

**Published:** 2011-12-20

**Authors:** Christopher F Sharpley, Vicki Bitsika, David R Christie

**Affiliations:** 1 Brain-Behaviour Research Group, University of New England, Armidale, New South Wales 2351, Australia; 2 Brain-Behaviour Research Group, Bond University, Gold Coast, Robina, Queensland 4229, Australia; 3 Premion, Tugun, Queensland 4221, Australia

## Abstract

**Background:**

Although it is well established that prostate cancer (PCa) patients are more likely to experience clinical depression than their age-matched non-prostate cancer peers, and that such depression can have negative effects upon survival, little is known about the underlying nature of the depressive symptomatology that these men experience. In particular, the incidence of melancholic symptoms of depression, which are signs of increased risk of suicide and resistance to treatment, has not previously been reported in PCa patients. The present study aimed to measure the incidence and nature of Melancholia in PCa depression.

**Method:**

A sample of 507 PCa patients in Queensland, Australia, completed anonymous and confidential questionnaires about their background, treatment status, and depression. Data were analysed to select depressive symptoms that were part of the definition of Melancholia *vs *those which were not. Regression was used to determine the links between Melancholia and overall depressive status, and factor analysis revealed the underlying components of Melancholia, which were mapped over time since diagnosis for 3 years.

**Results:**

Psychometric data were satisfactory. Melancholia significantly predicted depressive status for the most depressed subset of patients, but not for the total sample. Melancholia was factored into its components of Anhedonia and Agitation, and the first of these was more powerful in predicting Melancholia. Variability over the 3 years following diagnosis was noted for each of these two components of Melancholia.

**Conclusions:**

The strong presence of Melancholia in the depressive symptomatology of this sample of PCa patients suggests that some forms of treatment for depression may be more likely to succeed than others. The dominance of Anhedonia and Agitation over other symptoms of Melancholia also holds implications for treatment options when assisting these men to cope with their depression.

## Background

About 25 percent of prostate cancer (PCa) patients experience significant depression as a result of their illness, its diagnosis, or its treatment [1-3]. As well as emotional distress, depressed PCa patients also have significantly greater chances of admission to emergency treatment, hospitalization, outpatient visits and death [4]. Therefore, the ongoing investigation of the causes of depression among PCa patients and the efficacy of interventions aimed at reducing the intensity of that depression is clearly justified. Underlying that research is an understanding of the nature and structure of depression as it is experienced by this patient group, particularly as regards one of the major forms of depression---melancholic depression [5], which may have implications for both assessment and treatment of depression in PCa patients [6].

Although the first records of the term "melancholia" date from the ancient Greeks (from *melas *(meaning black) and *khole *(bile)), it was used by Hippocrates (*Aphorisms*, section 6.23) to describe what is nowadays referred to as "general depression": "If a fright or despondency lasts for a long time, it is a melancholic affliction" [7] , rather than the subset of depressive symptoms which are grouped under the modern term "melancholia". Melancholia was also used during the Middle Ages by Avicenna, in his *Canon of Medicine*, who described it as a depressive mood disorder which was also called "acedia", meaning an absence of caring [8] , much like anhedonia in modern definitions of depression [5] . In the fifteenth century, Robert Burton [9, p. 32] reported melancholy, its causes, symptoms and remedies in great detail, describing a typical sufferer as "we call him melancholy, that is dull, sad, sour, lumpish, ill-disposed, solitary, any way moved, or displeased". Samuel Johnson, in his description of *Rasselas, Prince of Abisinnia *[10] , referred to "melancholy" as containing a strong dose of unwarranted guilt, thereby linking the disorder with those who were prone to superstition. During the 18^th ^century, this focus upon the personality characteristics of those who suffered from melancholy was replaced by an emphasis upon more physiological bases, principally low energy and slowed circulation [11] , and the modern term "depression" was eventually used in place of melancholia. The latter term was then adopted to describe a particular subset of depressive symptoms.

Melancholic patients have been characterised as suffering from extreme and persistent anhedonia, plus psychomotor retardation or agitation, excessive guilt or hopelessness, suicidal features, and appetite disturbances or weight changes, all of which distinguish patients with melancholic depression from those with more general distress [6,12,13]. Of interest, patients with predominantly melancholic symptoms are not necessarily more depressed than others, with at least 50% of people who score in the upper half of the severity range for depression not showing the symptoms of melancholia [14]. These behavioural symptoms of melancholia have biological substrates that contribute to them, including dysfunction of the hypothalamic-pituitary-adrenal axis (which influences emotions and the sympathetic nervous system and may produce guilt and hopelessness [15]), the thyroid axis (which may contribute to psychomotor abnormalities, weight loss and sleep disturbances [16]), rapid eye movement patterns during sleep (which may be a result of altered circadian rhythms and thus contribute to sleep disturbances [17]), and left dorsolateral prefrontal cortex activity (which is linked with mood disorders [18]).

These concomitant biological factors which may occur before or alongside the psychological symptoms of depression may be potentially compounded by the physiological symptoms of cancer in general (or its treatment), leading some researchers to opt for the use of scales which omit somatic symptoms (e.g., the Hospital Anxiety and Depression Scale). For example, one study which used the same depression-screening scale as was used in the present study reported a factor structure for that scale which broke total scores into cognitive, depressed mood, and two somatic components among a sample of 1,109 patients with a range of cancers (although PCa was not mentioned in the sample description) [19] , suggesting that the "neurovegetative symptoms of depression were separable from the cognitive/ideational symptoms, with the latter being more reliable for diagnostic purposes" (p. 126), and that some somatic symptoms might be attributable to cancer and its treatment as much as to depression. However, another study with a mixed cancer sample (5% of the sample were PCa patients) and which used an abbreviated form of the same depression-screening scale found that this (somatic item-free) abbreviated scale correlated .91 with the entire (somatic item-inclusive) scale [20] , thus challenging the argument that all somatic items need to be excluded from assessment of depression among cancer patients.

While there is some validity in the argument proposed regarding deletion of somatic items from depression screening of mixed cancer patients due to the potential range of cancer-related physiological symptoms which might be confounded with depression symptomatology, the specific cancer-related symptoms that have been reported in PCa patients narrows the range of somatic symptoms that may need to be excluded. For example, several major reviews of specific PCa-related symptoms refer to: incontinence and impotence, problems with urinary control, sexual and bowel function [21] , tumor flare, hot flashes, and gynecomastia where antiandrogens are used [22] . Radiation-induced proctitis and bleeding have also been reported in PCa patients [23] . While these can cause significant discomfort, and may lead to some of the behavioural symptoms of melancholia listed at the commencement of this paragraph, they do not readily overlap with the somatic symptoms of depression as defined via the DSM-IV-TR, and it may be that scales which screen for depression and which include somatic symptoms can be delivered with some degree of confidence as long as the depression symptoms assessed are not those reported (above) that arise from PCa itself or its treatment.

Some data suggest that depressed persons who exhibit melancholic features are more likely to attempt violent suicide [24], although that outcome may be more related to some of the specific symptoms of melancholic depression such as guilty feelings, anhedonia and loss of interest rather than the complete group of symptoms that comprise melancholia [25]. In either case, the increased likelihood of suicide in PCa patients who exhibit melancholic features of depression lends urgency to investigations of the presence of melancholia among this patient group. As an additional impetus to such investigations, there is a longstanding clinical belief that patients with melancholic depression respond better to pharmacological treatments than to psychotherapy [26], presumably because of the powerful biological underpinnings to their depression. However, that position has been challenged as being more likely related to the biological correlates of melancholic depression rather than the psychological/behavioural symptomatology itself [17].

Despite the importance of understanding the extent of melancholia among depressed PCa patients, and how this might inform choice of treatments for those PCa patients whose depression exhibits melancholic features, a search of Google Scholar, PubMed and Science Direct in August, 2011, using the descriptors "prostate cancer, melancholic depression", failed to identify any study which had examined this issue within the PCa population. Therefore, the present study was designed to investigate the incidence of melancholic depression among a sample of PCa patients (using Frequencies analysis in SPSS), whether melancholic or nonmelancholic symptom clusters were stronger predictors of overall depressive status (using Linear Regression), whether melancholic symptomatology was more common or more intense among PCa patients who had severe depression than those with less severe depression (via MANOVA), and also to explore the structure of melancholic depression in a sample of PCa patients (via Factor Analysis), with a view to informing treatment recommendations for this patient group. Because of the exploratory nature of this study, *a priori *power analyses were not conducted, and *post hoc *examination of reported *B *values was done instead.

## Methods

### Sample

From 965 PCa patients in Brisbane, Australia, who were invited by letter to participate, 507 (52.22%) completed usable questionnaires. All participants had cancers limited to the primary site and regional draining lymph nodes using conventional staging investigations. Treatments included radiotherapy, plus hormone therapy and surgery when required.

### Measures

Background questionnaire: age, living situation, month and year of first diagnosis, treatments received and continuing, present status of their cancer.

Depression: The Zung Self-Rating Depression Scale (SDS) [27] is a standardised paper and pencil test of depression that has been used in studies of depression in PCa patients [e.g., 28, 29]. Having been developed on the basis of factor analytic studies of the syndrome of depression which underlie the DSM definition [5], the SDS includes items for all of the current DSM-IV-TR criteria for Major Depressive Episode (MDE). The SDS has 20 items and respondents are asked to indicate the frequency of each of the depressive symptoms contained in those 20 items "during the last two weeks" by answering in one of four possible ways: "None or a little of the time", "Some of the time", "Good part of the time", or "Most or all of the time". Raw scores range from 20 to 80, with higher scores being indicative of more severe depression. The SDS has demonstrated split-half reliability of .81 [27], .79 [30] and .94 [31]. Internal consistency (alpha) has been reported as .88 for depressed patients and .93 for non-depressed patients [32], and as .84 and .83 for previous Australian PCa samples [28,33]. The SDS has been shown to be superior to the MMPI Depression Scale and the Beck Depression Inventory for assessing depression in male psychiatric inpatients [32]. SDS scores of 40 or above indicate the presence of "clinically significant depression" [34, p. 335]. SDS raw scores were used in this study.

Melancholia: There are several scales which measure certain aspects of Melancholia such as anhedonia (e.g., the Chapman Physical and Social Anhedonia Scale [35] and the Snaith-Hamilton Pleasure Scale [36]), but are less able to tap other features. In addition to this issue of validity, there is also a potential measurement confound in using separate scales for depression and Melancholia, because some of the MDE symptomatology will be emphasised by repetition across two scales, and thereby alert participants to the relative importance of these features over non-melancholia features. Similarly, ethical considerations argue against applying two scales when one will suffice.

Therefore, because the SDS taps all the symptoms of MDE, it might also be used to measure the subset of items which are features of Melancholia. In order to identify which SDS items were also features of Melancholia, the first and second authors (both clinical psychologists who between them had over five decades experience in assessing and diagnosing MDE) allocated each of the 20 SDS items to the various criteria for melancholic depression as it is defined in the wider literature (described in the Introduction). That allocation was performed separately and blindly, and 11 of the SDS items were allocated with 100% agreement in this way (these 11 items are shown below in Table 1). The remaining 9 items were agreed to be not related to Melancholia, thus providing two subsets of SDS items: those related to melancholia (called "Melancholia") and those not related to melancholia ("Nonmelancholia"). The mean scores on each of these two subsets of SDS items may be used to clarify the underlying melancholic nature of the depression experienced by the men in this study. As for each of the 20 SDS items, the mean scores for each of these two subsets of SDS items had a range of possible scores from 1 to 4.

**Table 1:** SDS Melancholia items and their Beta weights (Standardised) as predictors of SDS total score

SDS item	Beta weight
20. I still enjoy doing the things I used to	.262
17. I feel I am useful and needed	.220
15. I am more irritable than usual	.197
18. My life is pretty full	.178
5. I eat as much as I used to	.161
13. I am restless and can't keep still	.152
6. I still enjoy sex	.145
2. Morning is when I feel best	.144
19. I feel others would be better off if I were dead	.091
9. My heart beats faster than usual	.087
7. I notice that I am losing weight	.078

Ethical approval for this study was obtained from the Wesley Human Research Ethics Committee, Brisbane. All participants gave written consent to take part in the study.

## Results

### Demographic data

None of the background variables showed any significant correlation with SDS scores.

### Psychometric data

Reliability (Cronbach's alpha) was satisfactory for the SDS (.84), allowing further examination of these data [37]. The mean SDS score was 34.92/80 (SD = 8.81), ranging from 20 to 66. The 5% trimmed mean was 34.58, only 0.34 less than the sample mean, thus discounting outlier effects. Skewness, kurtosis, boxplot inspection and examination of the Normal Q-Q Plot and Detrended Normal Q-Q Plot argued for the normality of these SDS data.

### Melancholia *vs *Nonmelancholia

Using the entire sample, the mean score for the Melancholia subset of SDS items was 1.778/4 (SD = 0.417), median = 1.700, ranging from 1.00 to 3.40; and the mean for Nonmelancholia SDS items was 1.721 (SD = 0.508), median = 1.667, ranging from 1.00 to 3.56. Reliability for the Melancholia subset of SDS items was .703, and skewness was .482, reflecting a slight clustering of scores to the left of the 1 to 4 point distribution, and kurtosis was 0.027, almost normal. Reliability for the Nonmelancholia subset of SDS items was .736, and skewness was .495, again indicating a slight clustering left of the mean, and kurtosis was -.335, reflecting a fairly flat distribution. None of the Q-Q Plots showed any departure from normality for these data

Using the Wilcoxen Signed Rank test, there was a significant difference between the distributions of these two subsets of SDS items (*M *Melancholia = 1.777, SD = .417; *M *Nonmelancholia = 1.721, SD = .508: *z *= 13.898, *p *< .001), with a small effect size (*r *= .17) for the total sample. To test if either of the two SDS subsets of items was a stronger predictor of SDS total score for the total sample, multiple regression was applied to the sample's scores on Melancholia and Nonmelancholia SDS items, and these were regressed against the total SDS score. The R square was .985, indicating that (as might be expected) almost all the variance in the SDS total score was predicted by these two subsets of SDS items; this result was significant (*F*(2,506) = 171.000, *p *< .001). Examination of the Beta weights (standardised coefficients) showed that Nonmelancholia made the strongest unique contribution to the SDS total score (*β *= .552, *p *< .001), followed by Melancholia (*β *= .518, *p *< .001), although both were significant predictors of the SDS total score.

Despite previous findings that Melancholia was not necessarily an indication of severe depression, there was a significant correlation between scores on the Melancholia subset of SDS items and SDS total score (*r *= .915, *p *< .001), accounting for 83.72% of the variance. Therefore, to explore this relationship further, Zung's recommended SDS cutoff score of 40 for "clinically significant" depression was applied to the entire sample, and 147 patients were identified as falling into this more severe depression category. The mean score for the Melancholia subset of SDS items for this subsample was 2.259/4 (SD = 0.296), median = 2.30, ranging from 1.60 to 3.40; the mean for Nonmelancholia SDS items was 2.314/4 (SD = 0.321), median = 2.333, ranging from 1.67 to 3.56. Using the Wilcoxen Signed Rank test, there was no significant difference between the distributions of these two subsets of SDS items (*z *= 1.594, *ns*) for this most depressed subsample. Multiple regression was applied to this subsample's scores on Melancholia and Nonmelancholia SDS items. The R square was .934, and this result was significant (*F*(2,146) = 1,022.000, *p *< .001). Examination of the Beta weights (standardised coefficients) showed that, in reverse to the finding for the total sample, Melancholia made the strongest unique contribution to the SDS total score (*β *= .631, *p *< .001), followed by Nonmelancholia (*β *= .576, *p *< .001), although (again) both were significant predictors of the SDS total score.

Finally, to test for the presence of significant differences between the PCa patients who had "clinically significant" depression versus those whose SDS score was below the recommended cutoff, MANOVA was used to compare the two subsamples on both Melancholia and Nonmelancholia subsets of SDS items. There was a significant main effect (*F*(2,504) = 470.00, *p *< .001, Wilks Lambda, partial eta squared = .651). Univariate testing indicated that the more severely depressed subsample had significantly higher mean scores for Melancholia (2.259) than the less severely depressed subsample (1.578: *F *= 613,145, *p *< .000); the same was true for Nonmelancholia (2.314 *vs *1.475: *F *= 669.015, *p *< .000).

As mentioned in the Introduction, melancholia represents a group of symptoms rather than a single symptom [6,12,13] (thus leading to the choice of SDS items to form a Melancholia subscale), and therefore the relative power of each of those symptoms to predict the total SDS score is of interest, and was investigated via multiple regression. The R square for the regression analysis was .889, indicating that a great deal of the variance in the SDS total score was predicted by the 11 SDS items that tapped melancholic features. This result was significant (*F*(11, 515) = 366.153, *p *< .001). The items and their Beta weights (standardised coefficients) are presented in descending order of predictive power in Table 1, and suggest that the first five SDS items shown in Table 1 (i.e., items 20, 17, 15, 18 and 5), plus item 6 might reflect anhedonia; items 13 and 9 may represent agitation; items 2 and 7 are discrete aspects of melancholia previously reported in the literature; and item 19 may indicate low self-esteem.

However, those groupings of SDS items with aspects of melancholia are based upon literal interpretation of each SDS item's content. While that is one way of grouping items for this purpose, another method is to apply factor analysis to the sample's responses to these items and then identify factors. There were many inter-item correlations greater than .3, the Kaisser-Meyer-Olkin measure of sampling adequacy was .784 (exceeding the recommended value of 0.6 ) and Bartlett's test of sphericity was significant (*p *< .001), thus justifying factor analysis with these data. Three components had eigenvalues greater than 1.0, but analysis of the screeplot via Catell's test and parallel analysis revealed that only the first two of these justified further examination. Together, these two components accounted for 42.428% of the variance (Factor 1 = 28.586%, Factor 2 = 13.842%). The component correlation matrix showed only a small correlation between these two components (*r *= .125) (accounting for less than 2% of the variance), arguing for their discreteness. When forced into a two-factor solution via Oblimin Rotation, the pattern matrix showed that SDS items 2, 5, 17, 18, 20 loaded on Factor 1, and SDS items 9, 13,15 and 19 loaded on Factor 2 (SDS 6 and 7 failed to load on either factor). From Table 1, the content of these items may be seen, and Factor 1 was labelled as "Anhedonia", and Factor 2 as "Agitation". The agreement between the factor analytic and content interpretation methods of identifying the components of the Melancholic subset of SDS items contributes to the reliability of that result, and justifies further investigation of these two components of Melancholia.

Two final analyses were undertaken using these factorial data. Although these analyses were largely exploratory, they were potentially informative for the purposes of planning treatments for PCa patients who exhibit melancholia. First, regression was used to determine the relative predictive power of each of the two components of Melancholia (i.e., Anhedonia, Agitation). The R square was .912, indicating that almost all the variance in the Melancholia score was predicted by these two subfactors, and this result was significant (*F*(2,510) = 2,624.000, *p *< .001). The Beta weight (standardised coefficients) for anhedonia was .766 (*p *< .001), and for Agitation it was .419 (*p *< .001), reflecting the stronger predictive power of the Anhedonia component for overall Melancholia.

**Figure 1:**
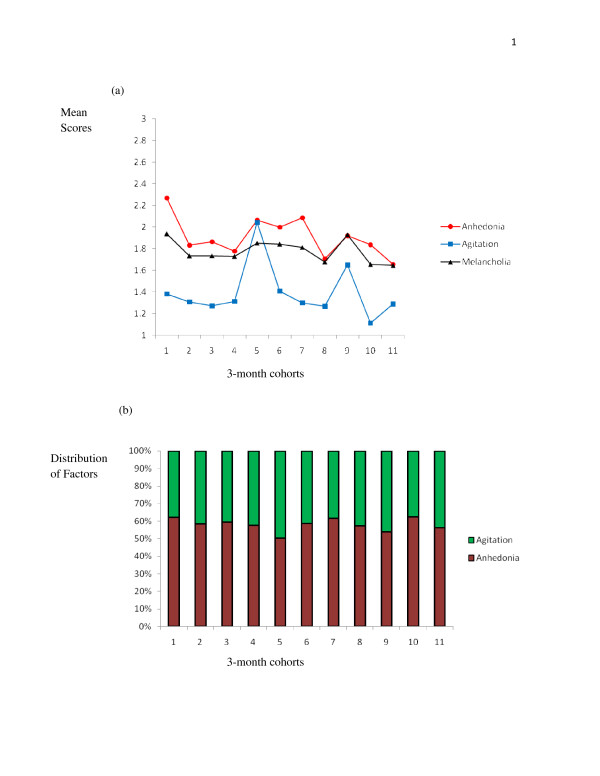
**Total Melancholia, Factor 1 (Anhedonia) and Factor 2 (Agitation): (a) incidence over 3-month cohorts, and (b) comparative weighting of Melancholia by Factors 1 and 2 over 3-month cohorts**.

The second analysis was performed to determine the presence of any variability in Melancholia, Anhedonia and Agitation over time. The sample was identified according to time since diagnosis, and classified into 3-month cohorts, ranging in subsample size from 10 for cohort 9 (i.e., 25 to 27 months after diagnosis) to 95 for cohort 2 (4 to 6 months after diagnosis). Although there were no significant correlations between time since diagnosis and SDS total score, the variabilities in total Melancholia scores (i.e., the mean of the 11 SDS items that comprise the Melancholia subset), the Anhedonia score (i.e., mean of items 2, 5, 17, 18, 20) and the Agitation score (mean of items 9, 13,15, 19) are shown in Figure 1(a) and 1(b). From Figure 1(a), it is apparent that Melancholia scores were relatively stable, although with a peak during the initial 3 months, and again at between 25 and 27 months after diagnosis. Anhedonia was highest immediately after diagnosis, then dropped for 12 months and increased during the period between 15 and 24 months after diagnosis. Agitation was fairly stable during the first 12 months after diagnosis but then increased dramatically at 15 months, decreased after that, and increased again at 27 months after diagnosis. Because these changes may have been due to current disease status (i.e., cancer still present, cancer in remission, cancer re-occurred after previous treatment) for various 3-monthly cohorts, MANOVA was conducted using current disease status over the 3-monthly cohorts as the Independent Variable and Melancholia, Anhedonia and Agitation as the Dependent Variables. There was no significant main effect (*F*(6,966) = 1.542, *p *= .161, Wilks Lambda, partial eta square = .009), nor any significant univariate effects. As noted by Stevens [38], with a sample size of over 100, "power is not an issue" (p. 6), and the *β *for this analysis was .600, also arguing that this nonsignificant outcome is not a result of a Type II error and it may be accepted that the lack of significant effects was not due to some of the sample having had unsuccessful treatment for their PCa. Figure 1(b) shows the relative distribution of the two Melancholia factors over the period examined, and reflects a consistent (although only slightly) higher incidence of Anhedonia over Agitation, with some variability during the 36-month period, and (most importantly for intervention planning) different peaks for the two factors of Melancholia at different times after diagnosis.

## Discussion

The allocation of SDS items into subsets to assess Melancholic and Nonmelancholic aspects of depression appears to have been accomplished with satisfactory internal consistency, and psychometric data allowed for use of these subscales to discuss the further incidence and distribution of Melancholia in this sample of PCa patients. Although the mean score for Melancholia was significantly higher than that for Nonmelancholia for the entire sample, Nonmelancholia was a stronger predictor of total SDS scores. There was a similar (but nonsignificant) difference in Melancholia and Nonmelancholia subset scores for those PCa patients whose SDS scores placed them in the "clinically significant" depression category, but Melancholia was a stronger predictor of total SDS score for this more depressed subsample. Although not consistently reported in the previous literature (none of which focussed on PCa patients), higher depression scores were significantly (and very meaningfully--over 80% of the variance) related to Melancholia. In addition, the most depressed patients were also distinguished by their depressive status being more a function of Melancholia than was the case for the less depressed PCa patients. Thus, depression in PCa patients appears to be closely related to Melancholia, making this population different to others in the literature and supporting the further exploration of the components of Melancholia that was conducted here.

The divisions of the Melancholia subset of SDS items to 'Anhedonia" and "Agitation" by content analysis and factor analysis produced markedly similar results, thus arguing that these two subfactors of Melancholia were robust in this sample, and produced a valuable extra metric for analysis of the overall Melancholia construct. Anhedonia had almost twice the predictive power for Melancholia as did Agitation, thereby reinforcing the finding reported above that, at least in this sample of PCa patients, melancholic depression was principally a function of loss of interest and/or pleasure in activities and sources in the patients' lives. That result was for the entire sample and provides some insight into the depressive states of these men. As well as being dominated by Melancholia in an overall sense, the key aspects of depression for these PCa patients were Anhedonia and Agitation, rather than the wider set of features that commonly describe Melancholia (such as excessive guilt or hopelessness, suicidal ideation, and appetite disturbances or weight changes). Of these two (i.e., anhedonia and agitation), loss of pleasure and/or interest is the predominant sub-feature. When subdivided according to time since diagnosis, there were changes in Melancholia and in the overall and relative incidence of Anhedonia and Agitation. These findings might be used to inform treatment planning with PCa patients.

For example, the relatively dominant position of Melancholia in the depressive profiles of this sample suggests that it might be profitably considered when developing psychosocial treatment regimes for men with PCa. If, as has been long believed, melancholic patients respond most effectively to medication rather than psychotherapy, then consideration of that avenue of ancillary treatment might be part of the overall PCa treatment protocols for those men who show signs of depression. This suggestion is reinforced by the finding that it was the Anhedonia aspect of Melancholia that was the strongest component, and that some previous data have indicated that anhedonia responds relatively poorly to psychotherapy compared to medication. Although self-help and self-guided treatments for PCa-linked depression are to be commended, they will be of relatively little efficacy with men who are anhedonic, and who are therefore biologically incapable of experiencing pleasure, either from behaviourally-induced activities or within the therapeutic bond of psychotherapy.

The second major finding that can inform treatment for PCa patients with Melancholic depression is that shown in Figures 1(a) and 1(b). Those data suggest that PCa patients exhibit different depressive profiles at different times following their diagnosis, arguing for variability in treatment protocols also. As reported in a previous study [39], the "one size fits all" treatment approach suffers in the face of changing depressive profiles during the three years after initial PCa diagnosis, and may need to be more closely aligned to the kinds of symptom variability shown in Figure 1. While Figure 1(a) can inform therapists about the 'danger' periods for Melancholia, Anhedonia and Agitation, Figure 1(b) may also provide some planning suggestions regarding the comparative attention to be given to the Anhedonia *vs *Agitation aspects of Melancholia in PCa patients.

There are several limitations to this study. First, a cross-sectional snapshot was used to gather data rather than a longitudinal design. While the latter may have been better able to show within-participant changes over time, there is also a potential reduction in the validity of self-report data when participants are asked to repeatedly comment on their psychological well-being. Second, the sample was from a single radiation oncology centre in one state in Australia, and generalisability to other types of treatment patient groups, different nationalities and cultures is therefore restricted. However, the size of the sample, and the robustness of the statistical findings argue for the validity of these results with this population. Finally, although a Danish sample of 278 men and 652 women with major depression showed that significantly more men than women fulfilled the criteria for melancholic depression [40] , as mentioned in the Introduction to this paper, no previous reports were found that focussed upon melancholia in PCa patients. The generalisability to PCa patients of data collected from severely depressed (but non-cancer) patients is not easily demonstrated, and therefore the present study may be seen as making a unique contribution to the literature regarding PCa patient depression.

## Conclusion

Melancholia played a major role in the depressive profiles of this sample of PCa patients, and Anhedonia was the principal subcomponent of that Melancholia. These findings suggest that PCa patients may have different depressive profiles to some previously reported non-PCa samples, and argue for consideration of specific PCa-related models of depression that incorporate specific treatment protocols. Most importantly, these data suggest that there is a reasonable proportion of PCa patients who have major depression (as previously reported), that there is a strong chance that they will also be melancholic, and that anhedonia will underlie their experiences of depression. Treatment options that favour pharmacological approaches may need to be given greater consideration for this segment of PCa patients who are depressed.

## Competing interests

The authors declare that they have no competing interests.

## Authors' contributions

DRHC recruited the patients and collected the data. He also reviewed and revised the ms. VB planned the data-collection, assisted with data analysis and helped draft the ms. She reviewed and revised the ms. CFS planned the study, developed the measures, performed the data analysis and drafted the first version of the ms. All authors approved the final version of the ms.

## Authors' information

CFS is Professor of neurophysiology at the University of New England, Australia, with particular interests in depression. VB is Professor of Clinical Psychology at Bond University, Australia, and researches functional analytic models of depression. DRHC is a Radiation Oncologist at Premion, and Associate Professor of Medicine at Bond University, Australia.

## Pre-publication history

The pre-publication history for this paper can be accessed here:

http://www.biomedcentral.com/1471-244X/11/201/prepub
